# Does intrauterine crowding affect the force generating capacity and muscle composition of the piglet front limb?

**DOI:** 10.1371/journal.pone.0223851

**Published:** 2019-10-10

**Authors:** Charlotte Vanden Hole, Chris Van Ginneken, Sara Prims, Miriam Ayuso, Steven Van Cruchten, Peter Aerts

**Affiliations:** 1 Laboratory of Applied Veterinary Morphology, Department of Veterinary Sciences, Faculty of Biomedical, Pharmaceutical and Veterinary Sciences, University of Antwerp, Wilrijk, Belgium; 2 Laboratory of Functional Morphology, Department of Biology, Faculty of Sciences, University of Antwerp, Wilrijk, Belgium; 3 Department of Movement and Sports Sciences, Faculty of Medicine and Health Sciences, University of Ghent, Ghent, Belgium; INIA, SPAIN

## Abstract

In the pig, intrauterine competition (IUC) greatly affects postnatal traits, such as birth weight, but also locomotor capacities. In a previous study, our group discovered a lower motor performance in piglets with a low birth weight and low vitality (L piglets), compared to piglets with a normal birth weight and normal vitality (N piglets). In order to explain the force deficit causing this reduced motor performance, in a subsequent study, we investigated whether this deficit in L piglets was caused by a lower force generating capacity (FGC) of the extensors of the hind limb and/or a lower number of type II (fast-twitch) fibers in m. vastus lateralis. L piglets had a lower absolute FGC, but surprisingly, a higher relative FGC (to birth weight) in the hind limb, compared to N piglets. In addition, we found no differences in fiber composition of m. vastus lateralis. In the present study, we assessed whether this higher relative FGC is a common feature for front and hind limb locomotor muscles of L piglets. To that end, the physiological cross-sectional area of the main extensor muscles of the front limb was calculated from their volume and fiber length, in order to calculate both the absolute and the relative FGC. By immunohistochemical staining of m. triceps brachii caput longum, the percentage of type II (fast-contracting) fibers could be determined. Similar to the results of the hind limb, we found a smaller absolute FGC, but a larger relative FGC in the front limb of L piglets, compared to N piglets. In addition, m. triceps brachii caput longum did not have a different muscle fiber composition in L and N piglets. As such, we can conclude that IUC affects the locomotor muscles in the front and hind limb in a similar way and that the observed force deficit in L piglets cannot be explained by a different force generating capacity or a lower percentage of type II muscle fibers.

## Introduction

In polytocous species, such as the pig (*Sus scrofa domesticus*), intrauterine competition (IUC) for uterine space and placental area leads to an increased variability in birth weights within a litter [1–4]. Because of genetic selection in modern breeding, the effect of IUC has become even more exacerbated. Consequently, more piglets with a low birth weight are born [4]. However, this low birth weight is only one of the ways IUC affects postnatal traits in pigs [5–11]. In a previous study, our group investigated the effect of IUC on the early development of locomotor capacities [12]. By studying spatio-temporal gait variables, we found a reduced motor performance (measured by self-selected speed) in piglets with a low birth weight and low vitality (L piglets), compared to piglets with a normal birth weight and normal vitality (N piglets) [12]. For L piglets, in order to increase their motor performance to the level of N piglets, they should increase their stride frequency, which implies a greater force generation and more rapid contractions [13]. However, their inability to increase motor performance (through an increased stride frequency) suggests L piglets experience a force deficit (i.e. are unable to produce enough force) [12].

The present study compares the force generating capacity (FGC) and muscle composition of the front limb in L and N piglets. In a previous report [14], our group investigated whether the force deficit in L piglets is caused by a lower FGC of the hind limb. We found that, at the level of the hind limb, L piglets indeed have a lower absolute FGC due to a smaller muscle volume. However, the FGC relative to body weight (BW) was higher in L piglets, suggesting that the relative physiological cross-sectional area (PCSA) of the L piglet hind limb is larger compared to that of N piglets.

In the present study, we investigate if this higher relative FGC of the hind limb is a common feature for other locomotor muscles of L piglets. In most quadrupeds, a degree of functional specialization of the legs can be observed, with (at least for non-primates) the hind legs being the most important for propulsion and acceleration (“rear-wheel drive”) [15–18]. Front legs are considered to be more important for the support of the center of mass (COM), given the fact that about 60% of the vertical impulse is applied through the thoracic limbs [18–22]. Given these (partially) different roles that are assumed by the leg pairs, they are both essential for coordinated locomotion.

To discover whether IUC affects locomotor muscles of the front and hind limb differently, we determine the muscle fiber length and volume and as such the PCSA for the main extensors of the front limb. From these measurements, the maximal isometric FGC (F_iso-max_, both absolute and relative to BW) is calculated. To check whether the conclusions from our study on the hind limb [14], that L piglets are not only more slender, but also smaller (with shorter legs), are also valid for the front limb, we also consider the length of the front limb in addition to body mass (BM) and body mass index (BMI).

However, as mentioned by Shahar and Milgram [23], calculation of the PCSA is a good estimate of a muscle’s ability to generate force, but does not take into account the presence of different fiber types. The two main muscle fiber types (type I and II) each have their own characteristics: type I are slow-twitch, oxidative fibers, while type II fibers are considered fast-twitch fibers that can range from having more oxidative to more glycolytic properties, depending on the subtype (a, b or x). Since their abundance is largely determined *in utero*, it is possible that muscle composition differs between L and N piglets (for extensive reviews on these topic see [10, 24]). As such, in our previous study on the hind limb [14], we investigated the composition of one of the major knee extensors, m. vastus lateralis. The composition of this muscle was not different for L and N piglets, but since results on muscle composition can differ greatly among muscles, we found it important to investigate the composition of an important extensor muscle of the front limb, m. triceps caput longum, by immunohistochemical fiber typing. More specifically, we expect a lower percentage of type II fibers in L piglets, causing the force deficit and associated lower performance observed in these piglets.

Specifically, this paper addresses the following questions:

- We hypothesize that L piglets are not only more slender (lower BM and BMI) compared to N piglets, but also overall smaller. This means that in addition to a lower BM and BMI, L piglets will have shorter front legs, compared to N piglets, as measured by the skeletal front limb length (SFLL)). This was also observed in the hind limb (measured by the skeletal hind limb length (SHLL)).
- We hypothesize that L piglets, because of their size, will have less voluminous muscles (and as such a smaller PCSA) leading to a lower F_iso-max_ both at birth and during early development, as was observed in the hind limb.
- To study the relative maximal FGC, F_iso-max_ was normalized to BW, thus yielding a dimensionless variable F’_iso-max_. Our study on the hind limb revealed a higher F’_iso-max_ because of a larger relative PCSA in L piglets, compared to N piglets. We hypothesize that IUC will affect the muscles of the front limb in a similar way. As such, we expect a higher relative FGC in the front limb of L piglets, compared to N piglets. This would also imply that the force deficit in L piglets is not caused by a lower FGC.
- We hypothesize a lower percentage of fast-contracting (type II) fibers in the front limb muscles of L piglets, compared to N piglets. As a case-study, we determined the muscle fiber composition in m. triceps caput longum and we calculated three ratios: type II muscle fiber to total muscle fiber (F_type II_/ F_total_), type II muscle fiber to total muscle tissue (F_type II_/ T_total_) and other (non-muscle fiber) to total muscle tissue (T_other_/T_total_).

## Material and methods

### Selection

Institutional and national guidelines for the care and use of animals were followed and all experimental procedures involving animals were approved by the Ethical Committee of Animal Experimentation, University of Antwerp, Belgium (approval number 2015–26).

Thirty-two piglets (Topigs 20 x German Piètrain) were selected from 10 litters in a local farm in October 2016. The same selection procedure was followed as in Vanden Hole et al. [12, 14]. To reduce the number of experimental animals, the piglets selected for Vanden Hole et al. [14] were also used in this study. The average duration of farrowing among the 10 selected litters was 4h53 ± 3h38 (mean ± SD, here and throughout).

From each litter, two to six piglets were selected in pairs of L and N piglets that were sex-matched (both being male or both being female). Only piglets that showed no apparent signs of diarrhea, respiratory distress or moribund weakness were selected. Piglets that were included in the study were ear-notched upon selection and remained with the sow for the entire studied period. The mean number of piglets born per litter was 18.2 (± 4.2). Table 1 contains an overview of the selected piglets. To determine whether a piglet was included in the study and whether it was classified as an L or N piglet, all piglets from the abovementioned 10 litters were weighed in order to calculate the mean BM per litter. The mean birth weight and number of piglets per litter can be found in Table 2. In addition, each piglet received a vitality score between 0–4, based on respiration (0–2, no to steady respiration) and movement (0–2, no movement to taking a few steps). Piglets that had a BM at birth that was within the limits of the mean BM at birth of the entire litter ± 1 SD, combined with a normal vitality score (3 or 4 out of 4) were classified as N piglets. Piglets with a BM at birth that was lower than the mean BM at birth of the litter– 1 SD, combined with a low vitality score (1 or 2 out of 4), belonged to the L piglet category. This selection procedure allowed us to focus on within-litter variation in birth weight and vitality, which is relevant in the context of IUC and competition. The mean BM at birth of N piglets (n = 17) was 1.37 kg (± 0.29), while that of L piglets (n = 15) was 0.79 kg (± 0.26). The BM at birth ranged from 0.94 to 1.9 kg in N piglets and 0.32 to 1.3 kg in L piglets. Same as in Vanden Hole et al. [14] (+ Vanden Hole et al., submitted), 4 time points were included, all within the frame of early development: 0, 4, 8 and 96 h after birth. For an elaborate explanation regarding these time points, we refer to Vanden Hole et al. [12, 14, 25]. In a nutshell, 96 h was used as a control age, while 0, 4 and 8 h proved crucial time points in the early locomotor development of the piglet [12, 25].

**Table 1:** Selected piglets, including category (N or L piglet), age (0, 4, 8 and 96 h) and sex.

Age	N piglets		L piglets		Total
	Male	Female	Male	Female	
**0 h**	2	3	1	3	9
**4 h**	2	2	2	2	8
**8 h**	2	2	2	2	8
**96 h**	2	2	1	2	7
**Total**	8	9	6	9	32

**Table 2:** Mean birth weight and number of piglets per litter (± SD).

Litter	n	Mean birth weight (kg) ± SD
**1**	20	1.08 ± 0.32
**2**	13	1.52 ± 0.12
**3**	22	1.26 ± 0.29
**4**	16	1.61 ± 0.22
**5**	18	1.37 ± 0.32
**6**	22	1.16 ± 0.27
**7**	26	0.82 ± 0.18
**8**	15	1.22 ± 0.24
**9**	15	1.51 ± 0.32
**10**	15	1.42 ± 0.23

### Sampling

Prior to euthanasia, the selected piglets were deeply anesthetized with a combination of Zoletil 100^®^ (Tiletamine 50 mg/ml, Zolazepam 50 mg/ml) and Sedaxyl^®^ (Xylazine hydrochloride 20 mg/ml), in a dosage of 0.22 ml/kg BM (administered intramuscularly). The actual euthanasia of the anesthetized animals took place by transecting the jugular veins and carotid arteries.

Immediately after euthanasia, the right front limb was separated from the rest of the body and frozen at -18°C awaiting dissection. In the pig, the thoracic limb is attached to the trunk via a synsarcosis (i.e. only muscle attachment with no bony articulation). To separate the front limb from the trunk, these attaching muscles were cut. Consequently, none of these muscles remained intact and as such they could not be included in the analysis.

From the left front leg, a tissue sample was taken from the m. triceps brachii caput longum for immunohistochemical fiber typing. The m. triceps brachii caput longum was chosen because of its function as an extensor of the elbow. The samples were fixated for 24 h in 4% paraformaldehyde solution (in 0.01 M phosphate-buffered saline solution (PBS), pH = 7.4) at room temperature (± 21°C). After rinsing with PBS, these tissue samples were further processed for paraffin embedding.

### Muscle dissection

Before dissection, the right front limbs were gently defrosted (in a sealed plastic bag) by submerging them in water of ± 38°C. For further dissection we selected the most important extensors of the front limb, because they are the main contributors to force generated for both the support of the body and forward movement: m. supraspinatus, m. triceps brachii (consisting of the caput accessorium, lateralis, longum and medialis) and m. extensor carpi radialis. The m. supraspinatus, m. triceps brachii and m. extensor carpi radialis are extensors of the shoulder, elbow and carpal joint, respectively. In addition, m. biceps brachii was selected because it is responsible for the synchronization of movements between the shoulder and the elbow.

Similar to Vanden Hole et al. [14], we decided against including the extensors of the digits (m. extensor digitorum communis and m. extensor digitorum lateralis), because they were too small for an accurate macroscopic measurement of fiber length (cf [26]). As such, including them would have introduced a greater error to the dataset, while (because of their small size) their contribution to the total FGC would have been limited, compared to other (larger) extensors.

After removal, the abovementioned muscles were stored in PBS to prevent them from desiccating and weighted individually (Sartorius BP 210 S, d = 0.1 mg).

### Fiber length and force calculations

The protocol for determining fiber length and calculating the FGC was identical to that described in Vanden Hole et al. [14]. As such, we refer to that study for more detailed information and formulas. In brief, muscles were cut along the line of the tendon and photographed. From these photos, 5 different fiber lengths per muscle (bundle) were measured with ImageJ (Rasband, W.S., ImageJ, U.S. National Institutes of Health, Bethesda, M.D. USA), from which the mean fiber length was calculated.

**Fig 1:**
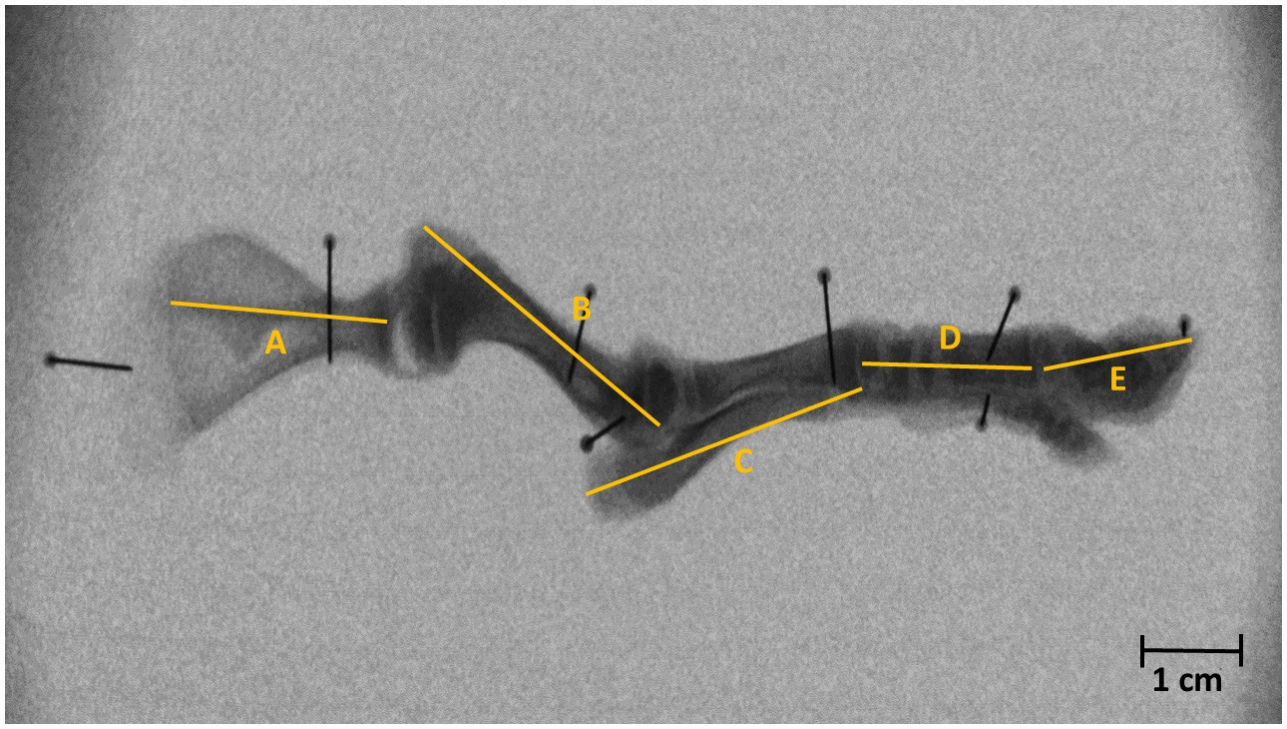
Skeletal front limb length (SFLL). A. Scapula. B. Humerus. C. Radius/ulna. D. Carpals/metacarpals. E. Phalanges. Category = N piglet, Age = 96 h, Sex = female.

After dissection, the remaining skeletal structures were X-rayed (settings: 45 kv, 30 mv, 150 ms) using 3D^2^YMOX (3-dimensional dynamic morphology using X-rays). The length of the scapula, humerus, radius/ulna, carpals/metacarpals and phalanges were measured with ImageJ (Rasband, W.S., ImageJ, U.S. National Institutes of Health, Bethesda, M.D. USA) and summed (Fig 1). The advantage of using X-rays to determine the bone length, was that the detailed picture allowed for an accurate measurement using anatomical landmarks (Table 3) and that bone and cartilage could be more easily distinguished from each other. The cartilage from the scapula was not included in the SFLL. This SFLL was combined with BM in order to calculate the BMI.

**Table 3:** Landmarks used for determination of bone lengths.

Bone	Proximal landmark	Distal landmark
**Scapula**	Most proximal end of spina scapulae	Cavitas glenoidalis
**Humerus**	Tuberculum majus	Capitulum (trochlea)
**Radius/ulna**	Tuber olecrani	Processus styloideus lateralis
**Carpals/metacarpals**	Most cranial end of proximal carpal bone	Most distal end of os metatarsale IV
**Phalanges**	Most proximal end of proximal phalanx	Most distal end of distal phalanx

From the muscle volume and mean fiber length, the PCSA per muscle was calculated in order to estimate F_iso-max_. F_iso-max_ was normalized to body weight (BW = BM x g; g = ms^-2^) to obtain F’_iso-max_. The values for F_iso-max_ and F’_iso-max_ per muscle were summed to get an approximation of the total F_iso-max_ and F’_iso-max_ of the front limb.

### Immunohistochemistry and image analysis

**Fig 2:**
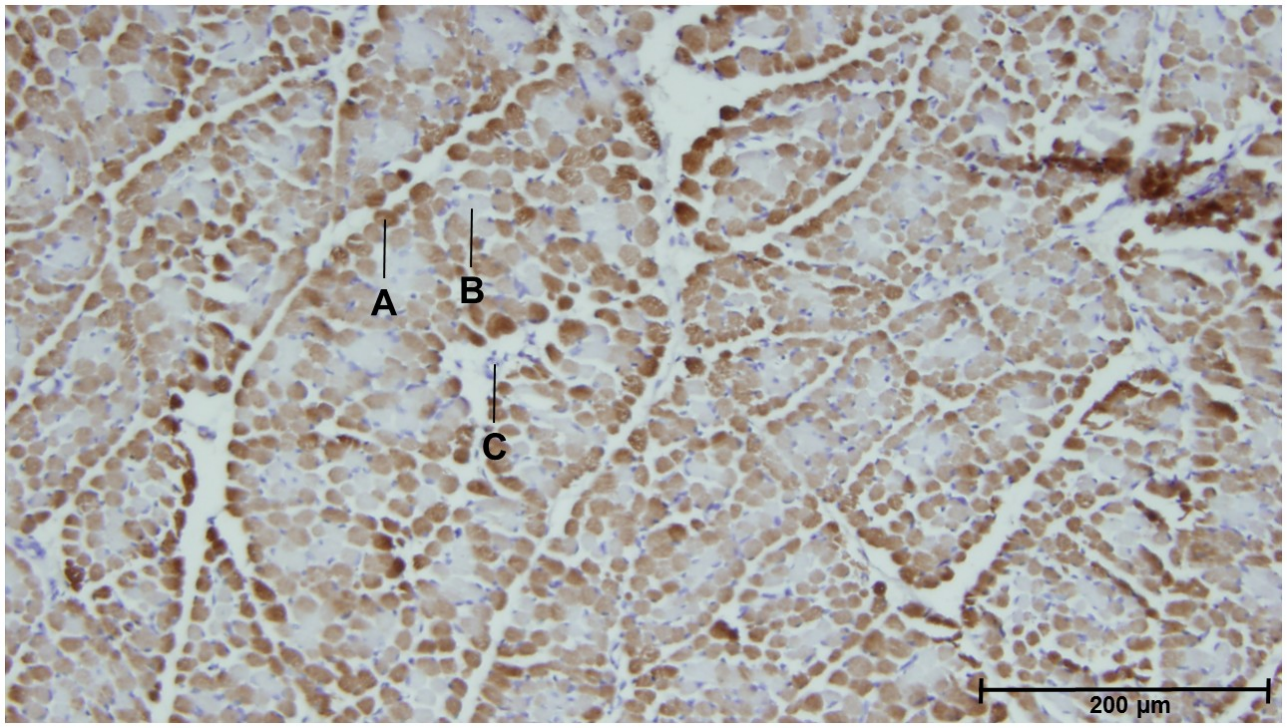
Type II staining of m. triceps brachii caput longum. A. Type II fiber (stained). B. Type I fiber (not stained). C. Connective tissue. Category = N piglet, Age = 96 h, Sex = female.

The muscle tissue was immunohistochemically stained for type II muscle fibers (see Fig 2) with a rabbit polyclonal anti-MYH1 antibody (Proteintech, Rosemont, IL, USA), using a protocol identical to that described in Vanden Hole et al. [14]. We refer to this paper for the protocol and the associated image analysis. Before commencing the study, a negative control was performed by applying the protocol (mentioned later in this section), with exclusion of the primary antibody. This was done on a subsample of the selected piglets (representing the different groups).

The number of type II fibers (F_type II_, stained), all muscle fibers (F_total_, stained + non-stained muscle fibers) as well as unstained, non-muscle fiber tissue (T_other_, such as connective tissue, fat, nerves and capillaries) were counted. From here on out, we will refer to the total muscle tissue as T_total_ (F_total_ + T_other_). From these data, three ratios were calculated: F_type II_/ F_total_; F_type II_/ T_total_ and T_other_ /T_total_.

### Statistics

To evaluate the effect of birth weight/vitality category (L or N piglet) and age (0, 4, 8 and 96 h) and sex on each of the outcome variables, linear mixed models were fitted using JMP^®^ Pro 13 (SAS Institute Inc., Cary, NC, USA). As such, the fixed effects of the starting model consisted of category, age, sex and their interactions. Because observations between littermates are not independent, the sow was added as a random factor to the model. Because of the insemination procedure on the farm (i.e. two inseminations with a mixture of semen from different boars) it was impossible to determine an individual boar per litter. As such, no random effect for the boar was included in the model. This starting model was gradually simplified using stepwise backwards modelling, during which all non-significant fixed effects were removed step-by-step. If necessary to meet normality and/or homoscedasticity assumptions, variables were transformed. An effect was considered statistically significant if *p* ≤ 0.05. *Post-hoc* analysis with Tukey’s correction was used to compare different age groups. All values are indicated as mean ± SD.

## Results

The significance of the predictor variables and their interactions on the different outcome variables can be found in Table 4. All relevant means ± SD’s are reported in Table 5. For calculations of F_iso-max_ and F’_iso-max_ per individual muscle, as well as more detailed information on means (± SD) across different groups, we refer to S1 and S2 Tables.

**Table 4:** The significance (*p*-values) of the predictor variables and their interactions on the different outcome variables. Significant effects (*p* ≤ 0.05) in bold.

Variable	Age	Cat (L/N)	Sex	Age*Cat	Age*Sex	Cat*Sex
**BM**	**< 0.0001**	**< 0.0001**	0.2036	0.2065	0.9905	0.9461
**SFLL**	**0.0003**	**< 0.0001**	**0.0403**	0.1291	0.2049	0.5901
**BMI**	**< 0.0001**	**< 0.0001**	0.1823	0.3201	0.8618	0.8247
**F_iso-max_**	0.1324	**0.0001**	0.8544	0.4036	0.2142	0.6747
**F’_iso-max_**	**< 0.0001**	**0.0006**	0.3814	0.3877	0.2002	0.4550
**F_type II_/ F_total_**	**0.0148**	0.5324	0.2101	0.9613	0.2319	0.6022
**F_type II_/ T_total_**	**0.0049**	0.7604	0.5420	0.9019	0.4252	0.8453
**T_other_/T_total_**	0.8539	0.4885	0.0983	0.7330	0.3561	0.2618

**Table 5:** Means ± SD for L and N piglets across ages. Values without a common superscript letter differ significantly (linear mixed models, *p* ≤ 0.05). *Post-hoc* analysis with Tukey’s correction was used to compare different age groups.

Category	L piglets				N piglets			
Age	0 h	4 h	8 h	96 h	0 h	4 h	8 h	96 h
**BM (kg)**	0.65 ± 0.28^**a**^	0.68 ± 0.21^**a**^	0.86 ± 0.22^**a**^	1.44 ± 0.21^**b**^	1.23 ± 0.36^**c**^	1.35 ± 0.23^**c**^	1.43 ± 0.20^**c**^	2.16 ± 0.38^**d**^
**SFLL (m)**	0.16 ± 0.03^**a**^	0.15 ± 0.02^**a**^	0.18 ± 0.01^**a**^	0.20 ± 0.02^**b**^	0.20 ± 0.02^**c**^	0.20 ± 0.02^**c**^	0.20 ± 0.01^**c**^	0.22 ± 0.01^**d**^
**BMI (kg/m^2^)**	23.4 ± 2.8^**a**^	28.3 ± 3.8^**a**^	26.8 ± 4.4^**a**^	35.1 ± 4.4^**b**^	29 ± 5.5^**c**^	33.5 ± 3.1^**c**^	34.7 ± 2.8^**c**^	44.1 ± 6.5^**d**^
**F_iso-max_ (N)**	175 ± 83^**a**^	141 ± 31^**a**^	167 ± 19^**a**^	229 ± 15^**a**^	249 ± 68^**b**^	243 ± 31^**b**^	232 ± 19^**b**^	290 ± 20^**b**^
**F’_iso-max_**	28 ± 6^**a**^	22 ± 5^**a,b**^	20 ± 4^**b**^	16 ± 3^**b**^	21 ± 2^**c**^	18 ± 2^**c,d**^	17 ± 3^**d**^	14 ± 2^**d**^
**F_type II_/ F_total_ (%)**	59.2 ± 3.2^**a**^	60.2 ± 1.6^**a,b**^	61.5 ± 2.2^**a,b**^	62.9 ± 2.2^**b**^	58.3 ± 3.4^**a**^	59.1 ± 2.9^**a,b**^	60.9 ± 1.7^**a,b**^	63.2 ± 4.2^**b**^
**F_type II_/ T_total_ (%)**	55.6 ± 2.5^**a**^	56.7 ± 1.6^**a,b**^	58.6 ± 2.8^**a,b**^	59.4 ± 1.6^**b**^	55.1 ± 3.8^**a**^	56.1 ± 2.3^**a,b**^	57.7 ± 1.2^**a,b**^	60.4 ± 4^**b**^
**T_other_/T_total_ (%)**	5.9 ± 1.1^**a**^	5.8 ± 1.5^**a**^	4.7 ± 2.9^**a**^	5.5 ± 1.4^**a**^	5.5 ± 1.3^**a**^	5 ± 0.7^**a**^	5.2 ± 1.8^**a**^	4.3 ± 0.6^**a**^

The data for BM are the same as in Vanden Hole et al. [14]. L piglets have a significantly lower BM than N piglets (, with both groups having a higher BM at 96 h, compared to 0, 4 and 8 h (*p* < 0.0001).

The mean SFLL was significantly shorter in L piglets compared to N piglets, across all time points. For both L and N piglets, the SFLL was shorter at 0 (*p* = 0.0007), 4 (*p* = 0.0008) and 8 h (*p* = 0.0045), compared to 96 h. In addition, females had a longer SFLL than males.

The BMI of L piglets was lower than that of N piglets. Both L and N piglets had a higher BMI at 96 h, compared to 0 (*p* < 0.0001), 4 (*p* = 0.0064) and 8 h (*p* = 0.0049).

The F_iso-max_ was significantly lower for L piglets, compared to N piglets. Age and sex had no significant effect on F_iso-max_.

The F’_iso-max_ was higher for L piglets, compared to N piglets. At 0 h, F’_iso-max_ was higher than at 8 (*p* = 0.0355) and 96 h (*p* = 0.0003), for both L and N piglets.

For none of the investigated variables we noted a difference between L and N piglets. However, F_type II_/ F_total_ and F_type II_/ T_total_ were significantly higher at 96 h, compared to 0 h (in both L and N piglets). T_other_/T_total_ did not differ significantly among age groups.

## Discussion

### Does IUC affect the locomotor muscles of the front and the hind limb differently?

Combining our results on morphometrics (BM, BMI and SFLL) and the absolute FGC, we can state that L piglets are overall smaller, i.e. they have shorter legs and are more slender, with a lower muscle volume. These results are the same as those of the hind limb [14]. These shorter legs will affect motor performance, in the sense that shorter legs will contribute to a shorter stride length and step length in L piglets [12]. The lower absolute FGC points to a lower muscle volume in L piglets, which is consistent with their lower BM and BMI. As previously discussed [14], these findings make sense in light of forward propulsion, maintaining posture and gravitational load, but do not tell us much in terms of muscle development. It is fairly straightforward that a lower BM would lead to a lower muscle mass and hence a lower F_iso-max_.

However, to figure out whether the musculoskeletal system of L piglets is sufficiently developed in L piglets, given their size, we have to look at the relative (or normalized, to BW) FGC. Well in line with our findings on the hind limb, F’_iso-max_ was higher in L piglets, compared to N piglets. In other words, relative to BM, they have a larger PCSA. We have found no studies comparing the FGC of the front limb between low birth weight or intrauterine growth restricted (IUGR) piglets and normal birth weight piglets. However, with regard to the hind limb, Bauer et al. [27] and Wank et al. [28] did find evidence supporting an accelerated muscle development in IUGR piglets.

As mentioned in the study on the hind limb, the higher F’_iso-max_ for L piglets might also be the consequence of a non-linear relationship between F_iso-max_ and BM. If F_iso-max_ increased more slowly, compared to BM, it would make sense that piglets with a lower BM (L piglets) show a relatively higher FGC, compared to animals with a higher BM (N piglets). This would explain why L piglets and piglets of the ages 0–8 h have a higher F’_iso-max_, compared to N piglets and piglets of 96 h old, respectively. A linear regression showed that 70% of the variation in F_iso-max_ can be explained by BM (R^2^ = 0.70, *p* < 0.0001). As such, we can (in all likelihood) dismiss the abovementioned hypothesis and state that, given their BM, L piglets have larger PCSA than N piglets.

As such, our study indicates that the effect of IUC on locomotor muscles is the same in the front and hind limb and that the force deficit in L piglets is not caused by a lower FGC.

In spite of the same results regarding FGC in the front and hind limb with regard to N and L piglets, it is obvious that the FGC is lower in the front limb, compared to the hind limb (in both L and N piglets). Both the absolute and relative FGC are on average twice as high in the hind limb, compared to the front limb. This difference might reflect the functional specialization that exists between front and hind limbs, but alternatively it might be caused by our selection of extensor muscles. To verify this, we took a closer look at the total mass (including the skeleton), total limb muscle mass and selected muscle mass (only the selected extensors), both absolute and as a percentage of body mass (S3 Table). Not including the extrinsic muscles leads to an underestimation of these masses and percentages, especially in the front limb (larger extrinsic muscle mass [29]). All masses and mass percentages were significantly higher in the hind leg, compared to the front leg (paired t-test, all *p*-values < 0.0001). This is a common finding in quadrupeds, for example in the horse [30], the cheetah [31, 32] and the hare [33, 34]. As mentioned in the introduction section of this paper, this has to do with the “rear-wheel drive” principle in quadruped locomotion, where the hind limbs need to be able to produce more force for propulsion, while the front legs are more important for the support of the center of mass [15–22]. However, in pigs, the difference in muscle mass between front and hind limbs might even be exacerbated because of domestication and intense production. Certain economically valuable muscles in meat production, e.g. the muscles of the ham such as m. semitendinosus, have undergone a selection for lean growth [35], which causes them to be disproportionally larger.

### Is the composition of the muscle different for L and N piglets?

Similar to our results on m. vastus lateralis [14], we noted no difference in the composition of m. triceps caput longum in L and N piglets. As mentioned in Vanden Hole et al. [14], results on muscle composition in relation to birth weight or growth retardation in pigs have been diverse. Some studies have found no relation between birth weight and fiber composition [5, 36], while others have reported an increased percentage of type I fibers in IUGR piglets [27, 28, 37]. All these studies cover only muscles from the back or the hind limb, the latter comprising of m. semitendinosus, m. flexor digitalis superficialis and m. gastrocnemius. To our knowledge, the fiber composition of front limb muscles in relation to birth weight in pigs has not been studied until now. In addition, in spite of both groups showing no difference in type II and type I distribution, we have to keep in mind that there might be other differences between L and N piglets with regard to fiber composition. In pigs, three subtypes of type II fibers can be distinguished (IIa, IIb, IIx) [38], that range from having more oxidative properties (IIa) to more glycolytic (IIb), with IIx possessing intermediate properties [39]. Given these different properties, it is possible that, if L and N piglets would have a different composition with regard to these subtypes, this might affect force production as well [40, 41].

In contrast to our results on m. vastus lateralis, the composition of m. triceps caput longum changed with age. More specifically, we noted an increase in fast fiber percentage (both relative to total fiber (0 h: 58.70%, 96 h: 63.09%) and all muscle tissue (0 h: 55.31%, 96 h: 59.98%)). Since no new muscle fibers are formed after birth and a conversion of fast fibers to slow fibers takes place during the first 8 weeks after birth [42–48], the observed increase in fast fiber percentage is most likely explained by a disproportional volume increase in fast and slow fibers, as was also reported by Handel and Stickland [49] and Lefaucheur [50]. After birth, an increase in muscle volume takes place by hypertrophy and an increase in myofiber length [5, 50, 51]. Therefore, muscle metabolism as a whole becomes more glycolytic [50].

This statement seems to be supported when we compare our results to results by Ono et al. [52] and Solomon et al. [53], who determined the composition of the m. triceps brachii in older pigs (on pigs of 20 and 30 kg, respectively). Though the goal of these studies was different from ours, we can deduce mean percentages of type II fibers from their papers, which are about 81% and 87% (in pigs of 20 and 30 kg, respectively). However, these results might not be entirely comparable to ours, since they did not distinguish between the different parts of the m. triceps brachii. According to Elder et al. [54] results can differ greatly among muscle parts (with the medial part of the m. triceps brachii in humans containing considerably less type II fibers than the other parts).

The fact that the composition of m. triceps caput longum changes with age, while that of m. vastus lateralis does not [14], once again points out how each muscle is different and that generalizations are difficult. As Lefaucheur [50] states: ‘muscle type is the most important factor influencing fiber type composition within an animal’. For example, deep muscles that are more involved in posture will contain more type I fibers, while more superficial muscles that are heavily involved in forward propulsion generally contain more type II fibers [52]. As such, it looks as if different muscles undergo a different maturation/differentiation process. Our results imply that firm conclusions with regard to possible differences (at birth and during the subsequent growth process) in muscle composition between L and N piglets should include as many muscles as possible, as one (or two) muscle(s) can merely be regarded as a case-study on the topic.

Interestingly, over the years, there appears to have been a general increase (across muscles) in glycolytic fiber proportions [55]. From our studies on m. vastus lateralis [14] and m. triceps caput longum, we also see that type II fibers take up larger volumes compared to type I (around 90% and 60% of type II fibers, respectively). It has been suggested by Henckel [55] that this increase is due to selection for higher lean growth rate, because glycolytic fibers have a higher growth potential than oxidative fibers. However, to find out whether this selection for lean growth rate has implications for motor performance, a comparative study of muscle composition and motor performance between the wild boar and the domestic pig is recommended.

## Conclusion

Our results show a lower absolute FGC, but a higher relative FGC in the front limb of L piglets, compared to N piglets. This means that, given their BM, L piglets actually have a larger PCSA than N piglets. In addition, we found no difference with regard to the muscle composition of m. triceps caput longum between L and N piglets. As such, we can conclude that IUC affects the locomotor muscles in the front and hind limb in a similar way and that the observed force deficit in L piglets cannot be explained by a different FCG or a lower percentage of type II muscle fibers.

## Supporting information

S1 Table Calculations F_iso-max_ and F’_iso-max_ per individual muscle. (PDF)

S2 Table Means (± SD) by category, sex and age. (PDF)

S3 Table Total mass (including the skeleton), total limb muscle mass and selected muscle mass (only the selected extensors), absolute and as a percentage of body mass. (PDF)
