# Supplementary material for: Does intrauterine crowding affect the force generating capacity and muscle composition of the piglet front limb?
Source: PLoS One. 2019 Oct 10;14(10):e0223851. doi: 10.1371/journal.pone.0223851 (PMC6786600; doi:10.1371/journal.pone.0223851)
Supplement: S1 Table — (PDF) [file pone.0223851.s001.pdf]

m. supraspinatus

PIGLET	SOW	CATEGORY	GENDER	AGE (in h)	BM (in kg)	FIBER LENGTH (in m)	MUSCLE MASS (in kg)	PCSA (in m^2)	$F_{iso-max}$ (in N)	$F'_{iso-max}$
1	2264	L	M	0	0.525	0.015434	0.0025479	0.000156329	46.8987448	9.106110345
2	2264	N	M	0	0.94	0.020064	0.0048665	0.000229686	68.90592151	7.472392642
3	1954	N	F	0	1.37	0.022442	0.0059312	0.000250275	75.08243472	5.58661538
4	1819	N	F	0	1.62	0.02649	0.0090919	0.000325019	97.50570541	6.135444143
5	1997	L	F	0	0.795	0.006836	0.0036446	0.000504875	151.4625113	19.42088503
6	1997	N	M	0	1.458	0.019414	0.0061766	0.00030128	90.38404806	6.319245924
7	2264	N	M	4	1.14	0.020322	0.0048045	0.000223881	67.16439193	6.005722046
8	1954	L	F	0	0.955	0.017834	0.0041288	0.000219236	65.77069336	7.02037064
9	2105	L	M	4	0.545	0.014134	0.0019041	0.000127574	38.27207443	7.158408744
10	2264	L	M	4	0.7	0.0151	0.0031483	0.00019744	59.23201385	8.625602715
11	2105	N	M	4	1.22	0.022414	0.0052357	0.000221203	66.36096961	5.544774453
12	2353	L	F	0	0.315	0.01608	0.0010744	6.32727E-05	18.98179557	6.142677724
13	2353	N	F	0	0.77	0.018488	0.0030456	0.000155998	46.79939814	6.195559545
14	1954	N	M	8	1.35	0.021448	0.005118	0.000225969	67.79080906	5.118798585
15	1954	L	M	8	0.705	0.017858	0.002443	0.000129547	38.86404362	5.619398879
16	2264	N	F	8	1.2	0.021938	0.0062755	0.000270887	81.26595405	6.903326032
17	2264	L	F	8	0.65	0.01953	0.0037033	0.000179565	53.86962947	8.448150157
18	1819	L	M	8	1.1	0.022992	0.004482	0.0001846	55.37993452	5.132048422
19	1819	N	M	8	1.648	0.028998	0.0083271	0.000271933	81.57988168	5.046111661
20	2321	L	F	4	0.5	0.014548	0.0017881	0.000116392	34.91771752	7.118800719
21	2321	N	F	4	1.38	0.02379	0.0063972	0.000254643	76.39286943	5.642930862
22	1870	L	F	4	0.975	0.02023	0.0036165	0.000169289	50.78669168	5.309777222
23	1870	N	F	4	1.65	0.030916	0.0078628	0.000240841	72.25223185	4.46373409
24	1196	L	F	8	1	0.027	0.0039904	0.000139955	41.98653199	4.27997268
25	1196	N	F	8	1.52	0.0250408	0.0061751	0.000233524	70.05725746	4.698297754
26	2105	L	M	96	1.2	0.016312	0.0048408	0.000281026	84.30770431	7.161714603
27	1997	L	F	96	1.52	0.024544	0.005854	0.000225862	67.75864496	4.544144332
28	1997	N	F	96	2.63	0.036174	0.0120357	0.000315073	94.52183763	3.663594518
29	2105	N	M	96	2.265	0.032104	0.0108328	0.000319534	95.86032893	4.314214172
30	2342	L	F	96	1.605	0.031448	0.007146	0.000215182	64.5546183	4.099994494

31	2342	N	F	96	2.005	0.02517	0.0073251	0.000275592	82.67756528	4.203434598
32	2353	N	M	96	1.73	0.028526	0.0080451	0.000267071	80.12128489	4.720986895

m. biceps brachii

PIGLET	SOW	CATEGORY	GENDER	AGE (in h)	BM (in kg)	FIBER LENGTH (in m)	MUSCLE MASS (in kg)	PCSA (in m^2)	$F_{iso-max}$ (in N)	$F'_{iso-max}$
1	2264	L	M	0	0.525	0.007252	0.0004637	6.05502E-05	18.16505165	3.52702328
2	2264	N	M	0	0.94	0.014132	0.0008689	5.8224E-05	17.46720853	1.89420354
3	1954	N	F	0	1.37	0.013732	0.0012019	8.2884E-05	24.86519543	1.850130243
4	1819	N	F	0	1.62	0.013326	0.0013363	9.49599E-05	28.48796952	1.792575573
5	1997	L	F	0	0.795	0.008856	0.0006211	6.64141E-05	19.92421676	2.55473067
6	1997	N	M	0	1.458	0.011404	0.0012367	0.000102694	30.80806974	2.153961604
7	2264	N	M	4	1.14	0.009756	0.0009751	9.46484E-05	28.3945311	2.538989136
8	1954	L	F	0	0.955	0.009832	0.0007342	7.07145E-05	21.21435572	2.264422533
9	2105	L	M	4	0.545	0.007862	0.000418	5.03477E-05	15.10429916	2.825108092
10	2264	L	M	4	0.7	0.006546	0.0005986	8.65958E-05	25.97873788	3.783127695
11	2105	N	M	4	1.22	0.010442	0.0009712	8.80767E-05	26.42301196	2.207768249
12	2353	L	F	0	0.315	0.005144	0.0002373	4.36851E-05	13.10551569	4.241061338
13	2353	N	F	0	0.77	0.01383	0.0006049	4.14188E-05	12.42563926	1.644973888
14	1954	N	M	8	1.35	0.007548	0.001083	0.000135873	40.76185142	3.077876047
15	1954	L	M	8	0.705	0.008262	0.000478	5.47872E-05	16.43614797	2.376522433
16	2264	N	F	8	1.2	0.013726	0.0011838	8.16715E-05	24.50144384	2.081332301
17	2264	L	F	8	0.65	0.010832	0.000717	6.26825E-05	18.80476198	2.949072686
18	1819	L	M	8	1.1	0.011864	0.0009482	7.56841E-05	22.70524275	2.104090701
19	1819	N	M	8	1.648	0.017162	0.0013386	7.38616E-05	22.15849498	1.370610469
20	2321	L	F	4	0.5	0.007574	0.00043	5.37625E-05	16.12874187	3.288224642
21	2321	N	F	4	1.38	0.014362	0.0012475	8.22549E-05	24.67646631	1.822782602
22	1870	L	F	4	0.975	0.015132	0.0006659	4.16724E-05	12.50172722	1.307062623
23	1870	N	F	4	1.65	0.017662	0.0015729	8.4333E-05	25.29988625	1.563023893
24	1196	L	F	8	1	0.01075	0.0008389	7.38989E-05	22.16966173	2.259904356
25	1196	N	F	8	1.52	0.013032	0.0010302	7.48594E-05	22.45783107	1.506104879
26	2105	L	M	96	1.2	0.009662	0.0010854	0.00010638	31.91391769	2.711002182
27	1997	L	F	96	1.52	0.018772	0.0012448	6.2795E-05	18.83850222	1.263379354
28	1997	N	F	96	2.63	0.021582	0.0023683	0.000103916	31.17470577	1.208307879

29	2105	N	M	96	2.265	0.020354	0.0022624	0.000105258	31.5774429	1.421149428
30	2342	L	F	96	1.605	0.020398	0.0016071	7.4609E-05	22.38270909	1.421571166
31	2342	N	F	96	2.005	0.02307	0.0015541	6.37922E-05	19.13765418	0.972983147
32	2353	N	M	96	1.73	0.018596	0.0016204	8.25161E-05	24.75483486	1.458629266

m. triceps brachii caput longum

PIGLET	SOW	CATEGORY	GENDER	AGE (in h)	BM (in kg)	FIBER LENGTH (in m)	MUSCLE MASS (in kg)	PCSA (in m^2)	$F_{iso\text{-}max}$ (in N)	$F'_{iso\text{-}max}$
1	2264	L	M	0	0.525	0.02381	0.0027933	0.000111095	33.32848116	6.471235602
2	2264	N	M	0	0.94	0.024252	0.0056177	0.000219355	65.80642834	7.136273054
3	1954	N	F	0	1.37	0.025634	0.0072102	0.000266359	79.90763333	5.945641147
4	1819	N	F	0	1.62	0.030794	0.0091874	0.000282529	84.75861591	5.333346919
5	1997	L	F	0	0.795	0.02648	0.0037172	0.000132933	39.88001236	5.113510455
6	1997	N	M	0	1.458	0.029358	0.0076835	0.000247838	74.35153961	5.198325077
7	2264	N	M	4	1.14	0.027524	0.006313	0.0002172	65.16007517	5.82649956
8	1954	L	F	0	0.955	0.028628	0.0046975	0.000155386	46.61579731	4.975775047
9	2105	L	M	4	0.545	0.022056	0.0021153	9.082E-05	27.24598749	5.096089459
10	2264	L	M	4	0.7	0.02705	0.0038381	0.000134365	40.30940178	5.870016278
11	2105	N	M	4	1.22	0.030066	0.0058405	0.000183955	55.18635517	4.611082299
12	2353	L	F	0	0.315	0.01992	0.0011946	5.67897E-05	17.03689759	5.513291455
13	2353	N	F	0	0.77	0.025184	0.0033458	0.000125809	37.74266851	4.996580286
14	1954	N	M	8	1.35	0.033912	0.0066386	0.000185378	55.61352645	4.199307317
15	1954	L	M	8	0.705	0.023134	0.00288	0.00011789	35.3670709	5.113767382
16	2264	N	F	8	1.2	0.031252	0.007208	0.00021841	65.52307925	5.56601081
17	2264	L	F	8	0.65	0.022252	0.0039866	0.000169656	50.89685503	7.981942293
18	1819	L	M	8	1.1	0.030434	0.0062491	0.000194444	58.33319643	5.405726663
19	1819	N	M	8	1.648	0.038032	0.0092144	0.000229432	68.82959804	4.257444729
20	2321	L	F	4	0.5	0.01896	0.0019702	9.84029E-05	29.52088128	6.018528294
21	2321	N	F	4	1.38	0.032446	0.0072995	0.000213043	63.91301211	4.721078174
22	1870	L	F	4	0.975	0.033274	0.0038855	0.00011058	33.17410673	3.468371544
23	1870	N	F	4	1.65	0.025118	0.0095522	0.000360126	108.0377889	6.67456145
24	1196	L	F	8	1	0.022184	0.0041741	0.00017818	53.45401477	5.448931169
25	1196	N	F	8	1.52	0.029004	0.0056567	0.000184689	55.40673857	3.715779989
26	2105	L	M	96	1.2	0.032614	0.0057204	0.000166096	49.82871271	4.232816234

27	1997	L	F	96	1.52	0.027498	0.0063818	0.000219775	65.93248104	4.421675052
28	1997	N	F	96	2.63	0.0406444	0.0143682	0.000334763	100.4289644	3.892550257
29	2105	N	M	96	2.265	0.049466	0.01408	0.000269545	80.8636235	3.639284305
30	2342	L	F	96	1.605	0.028724	0.0091308	0.000301023	90.30696535	5.735578188
31	2342	N	F	96	2.005	0.0303518	0.0109014	0.000340121	102.0364076	5.187663238
32	2353	N	M	96	1.73	0.026474	0.0088961	0.000318212	95.46351652	5.624997291

m. triceps brachii caput lateralis

PIGLET	SOW	CATEGORY	GENDER	AGE (in h)	BM (in kg)	FIBER LENGTH (in m)	MUSCLE MASS (in kg)	PCSA (in m^2)	$F_{iso-max}$ (in N)	$F'_{iso-max}$
1	2264	L	M	0	0.525	0.0205	0.0008347	3.85578E-05	11.56735033	2.245978415
2	2264	N	M	0	0.94	0.02407	0.0017832	7.01552E-05	21.04656872	2.282361542
3	1954	N	F	0	1.37	0.027874	0.0026088	8.86294E-05	26.58880547	1.9783779
4	1819	N	F	0	1.62	0.033456	0.0030983	8.76972E-05	26.30914824	1.655475532
5	1997	L	F	0	0.795	0.029936	0.0013499	4.27016E-05	12.81047295	1.642589445
6	1997	N	M	0	1.458	0.034252	0.0024898	6.88358E-05	20.65075165	1.443807629
7	2264	N	M	4	1.14	0.02811	0.0017463	5.88294E-05	17.64880664	1.578125314
8	1954	L	F	0	0.955	0.030372	0.0017808	5.55236E-05	16.65708847	1.777979353
9	2105	L	M	4	0.545	0.022224	0.0007471	3.18341E-05	9.5502303	1.786275061
10	2264	L	M	4	0.7	0.027946	0.0012176	4.12592E-05	12.37776751	1.802500002
11	2105	N	M	4	1.22	0.03511	0.001817	4.90072E-05	14.7021698	1.228436173
12	2353	L	F	0	0.315	0.01961	0.0004255	2.05475E-05	6.164236707	1.994801776
13	2353	N	F	0	0.77	0.025906	0.0012469	4.55793E-05	13.67378038	1.810209617
14	1954	N	M	8	1.35	0.035558	0.0019745	5.25843E-05	15.77528264	1.191171717
15	1954	L	M	8	0.705	0.026738	0.0010898	3.8597E-05	11.57911111	1.674237622
16	2264	N	F	8	1.2	0.028504	0.0021201	7.04347E-05	21.13040753	1.794971758
17	2264	L	F	8	0.65	0.028184	0.0012519	4.20633E-05	12.61898272	1.978982627
18	1819	L	M	8	1.1	0.03242	0.0017002	4.96619E-05	14.89856149	1.380646974
19	1819	N	M	8	1.648	0.042238	0.002943	6.59816E-05	19.79448708	1.224385106
20	2321	L	F	4	0.5	0.024998	0.0007461	2.82636E-05	8.479087418	1.728662063
21	2321	N	F	4	1.38	0.040782	0.0025885	6.01057E-05	18.03171297	1.331952974
22	1870	L	F	4	0.975	0.031812	0.001337	3.97994E-05	11.93981974	1.248314879
23	1870	N	F	4	1.65	0.03486	0.0030899	8.39369E-05	25.18108147	1.555684148
24	1196	L	F	8	1	0.034852	0.0016073	4.36722E-05	13.10166757	1.335542056

25	1196	N	F	8	1.52	0.039598	0.0020359	4.86877E-05	14.60631047	0.979552985
26	2105	L	M	96	1.2	0.030526	0.0017303	5.36769E-05	16.10307607	1.367913359
27	1997	L	F	96	1.52	0.032032	0.0021968	6.49445E-05	19.48335755	1.306625728
28	1997	N	F	96	2.63	0.044012	0.00499	0.000107366	32.20970727	1.24842375
29	2105	N	M	96	2.265	0.042018	0.003746	8.44245E-05	25.32734889	1.139862639
30	2342	L	F	96	1.605	0.037026	0.0023731	6.06939E-05	18.20818172	1.156438482
31	2342	N	F	96	2.005	0.038806	0.0033281	8.12145E-05	24.3643497	1.238715124
32	2353	N	M	96	1.73	0.035908	0.0029993	7.90979E-05	23.72936013	1.398205213

m. triceps brachii caput medialis

PIGLET	SOW	CATEGORY	GENDER	AGE (in h)	BM (in kg)	FIBER LENGTH (in m)	MUSCLE MASS (in kg)	PCSA (in m^2)	$F_{iso\text{-}max}$ (in N)	$F'_{iso\text{-}max}$
1	2264	L	M	0	0.525	0.015604	0.000337	2.04517E-05	6.135518865	1.191305056
2	2264	N	M	0	0.94	0.025188	0.0007513	2.82459E-05	8.473777195	0.918925239
3	1954	N	F	0	1.37	0.026978	0.0010001	3.51051E-05	10.53151895	0.783612651
4	1819	N	F	0	1.62	0.020486	0.0014983	6.92592E-05	20.77777063	1.307419402
5	1997	L	F	0	0.795	0.0135024	0.0004978	3.49124E-05	10.47372723	1.342966326
6	1997	N	M	0	1.458	0.020594	0.0010834	4.98178E-05	14.9453283	1.044910102
7	2264	N	M	4	1.14	0.02006	0.0009104	4.29771E-05	12.89313877	1.15288184
8	1954	L	F	0	0.955	0.018582	0.0006161	3.13975E-05	9.419244919	1.005411181
9	2105	L	M	4	0.545	0.015224	0.000444	2.76179E-05	8.285362824	1.54969425
10	2264	L	M	4	0.7	0.013058	0.0004656	3.37654E-05	10.12963143	1.475117436
11	2105	N	M	4	1.22	0.02328	0.0009106	3.70408E-05	11.11225008	0.928481315
12	2353	L	F	0	0.315	0.012842	0.0002094	1.54412E-05	4.632349818	1.499069566
13	2353	N	F	0	0.77	0.014466	0.0005461	3.57487E-05	10.72459875	1.419780868
14	1954	N	M	8	1.35	0.024182	0.0008477	3.3196E-05	9.958806701	0.751976947
15	1954	L	M	8	0.705	0.011982	0.0004203	3.32174E-05	9.965231939	1.440884889
16	2264	N	F	8	1.2	0.02306	0.0009889	4.06096E-05	12.18289245	1.034904218
17	2264	L	F	8	0.65	0.015962	0.000597	3.54179E-05	10.62537732	1.666333775
18	1819	L	M	8	1.1	0.02229	0.000911	3.8703E-05	11.61089359	1.07597939
19	1819	N	M	8	1.648	0.029538	0.0012955	4.15329E-05	12.45987449	0.770703716
20	2321	L	F	4	0.5	0.02009	0.0003864	1.82135E-05	5.464048147	1.113975157
21	2321	N	F	4	1.38	0.021908	0.0011955	5.16753E-05	15.50258727	1.145133424
22	1870	L	F	4	0.975	0.019188	0.0005924	2.92362E-05	8.770870051	0.916999404

23	1870	N	F	4	1.65	0.031898	0.0014284	4.24055E-05	12.72165824	0.785942498
24	1196	L	F	8	1	0.029018	0.0006123	1.99817E-05	5.994515943	0.611061768
25	1196	N	F	8	1.52	0.02933	0.0008876	2.86577E-05	8.597309612	0.576567252
26	2105	L	M	96	1.2	0.022852	0.0009734	4.0337E-05	12.10108922	1.027955252
27	1997	L	F	96	1.52	0.024416	0.0009046	3.50847E-05	10.52541925	0.705873387
28	1997	N	F	96	2.63	0.03613	0.0018532	4.85725E-05	14.57174848	0.564789886
29	2105	N	M	96	2.265	0.03322	0.0018665	5.32065E-05	15.96194105	0.718370499
30	2342	L	F	96	1.605	0.02656	0.0014479	5.16234E-05	15.4870191	0.983611935
31	2342	N	F	96	2.005	0.026124	0.001611	5.83972E-05	17.51915689	0.890696647
32	2353	N	M	96	1.73	0.032878	0.0013996	4.0312E-05	12.09360777	0.712591715

m. triceps brachii caput accessorius

PIGLET	SOW	CATEGORY	GENDER	AGE (in h)	BM (in kg)	FIBER LENGTH (in m)	MUSCLE MASS (in kg)	PCSA (in m^2)	$F_{iso-max}$ (in N)	$F'_{iso-max}$
1	2264	L	M	0	0.525	0.014724	0.000126	8.10365E-06	2.431095799	0.472034522
2	2264	N	M	0	0.94	0.016572	0.0003842	2.19542E-05	6.586273671	0.714237933
3	1954	N	F	0	1.37	0.017902	0.000278	1.47055E-05	4.41164522	0.328254739
4	1819	N	F	0	1.62	0.019342	0.0003358	1.64405E-05	4.932154238	0.310350627
5	1997	L	F	0	0.795	0.012262	0.0003002	2.31838E-05	6.955153393	0.891806383
6	1997	N	M	0	1.458	0.016924	0.0003525	1.97239E-05	5.91716175	0.413701323
7	2264	N	M	4	1.14	0.015406	0.0003121	1.9184E-05	5.755210485	0.514620821
8	1954	L	F	0	0.955	0.017006	0.0002123	1.18218E-05	3.546542397	0.378558304
9	2105	L	M	4	0.545	0.009972	0.0001062	1.00851E-05	3.025516902	0.565892677
10	2264	L	M	4	0.7	0.016498	0.0002902	1.66572E-05	4.99716219	0.727706741
11	2105	N	M	4	1.22	0.018916	0.0003247	1.62551E-05	4.876523482	0.407456717
12	2353	L	F	0	0.315	0.010162	0.0000406	3.78341E-06	1.135021739	0.367303121
13	2353	N	F	0	0.77	0.016888	0.0001725	9.67268E-06	2.901804939	0.384156763
14	1954	N	M	8	1.35	0.02045	0.0003264	1.51145E-05	4.534340965	0.342382374
15	1954	L	M	8	0.705	0.011438	0.0001873	1.55069E-05	4.652056939	0.672646516
16	2264	N	F	8	1.2	0.021404	0.0003158	1.39718E-05	4.191548733	0.356060885
17	2264	L	F	8	0.65	0.016648	0.0002537	1.44309E-05	4.329280612	0.67894309
18	1819	L	M	8	1.1	0.016504	0.0003389	1.94455E-05	5.83364088	0.540602435
19	1819	N	M	8	1.648	0.026064	0.000357	1.29707E-05	3.891208354	0.240690124
20	2321	L	F	4	0.5	0.019052	0.0001293	6.42679E-06	1.928036665	0.393075773

21	2321	N	F	4	1.38	0.020938	0.0003643	1.64763E-05	4.942894172	0.365117979
22	1870	L	F	4	0.975	0.019724	0.0002226	1.06873E-05	3.206177062	0.335207618
23	1870	N	F	4	1.65	0.02189	0.0005981	2.5874E-05	7.762209809	0.479548377
24	1196	L	F	8	1	0.015626	0.0001853	1.12296E-05	3.368875301	0.343412365
25	1196	N	F	8	1.52	0.017966	0.0002205	1.16223E-05	3.486699625	0.233830921
26	2105	L	M	96	1.2	0.01515	0.0003069	1.91832E-05	5.754950495	0.488867694
27	1997	L	F	96	1.52	0.020294	0.0002522	1.17683E-05	3.530488187	0.236767543
28	1997	N	F	96	2.63	0.026722	0.000632	2.23967E-05	6.719012594	0.260423817
29	2105	N	M	96	2.265	0.032898	0.000591	1.70119E-05	5.103584633	0.229687895
30	2342	L	F	96	1.605	0.026942	0.0003967	1.39434E-05	4.183017728	0.265671924
31	2342	N	F	96	2.005	0.024072	0.0004432	1.74351E-05	5.23052056	0.265926446
32	2353	N	M	96	1.73	0.023238	0.000452	1.84194E-05	5.52582369	0.325598139

m. extensor carpi radialis

PIGLET	SOW	CATEGORY	GENDER	AGE (in h)	BM (in kg)	FIBER LENGTH (in m)	MUSCLE MASS (in kg)	PCSA (in m^2)	$F_{iso-max}$ (in N)	$F'_{iso-max}$
1	2264	L	M	0	0.525	0.007904	0.0007136	8.54956E-05	25.64869341	4.980087066
2	2264	N	M	0	0.94	0.01386	0.0013091	8.94429E-05	26.8328578	2.909846422
3	1954	N	F	0	1.37	0.012986	0.0019043	0.000138866	41.65981197	3.099757581
4	1819	N	F	0	1.62	0.012072	0.0029117	0.000228404	68.52116468	4.311622348
5	1997	L	F	0	0.795	0.00888	0.0010154	0.000108283	32.48489967	4.16529144
6	1997	N	M	0	1.458	0.01184	0.0019737	0.000157858	47.35728271	3.311008105
7	2264	N	M	4	1.14	0.011854	0.0014609	0.000116706	35.01167615	3.130682632
8	1954	L	F	0	0.955	0.009526	0.0013045	0.000129679	38.9036942	4.152584359
9	2105	L	M	4	0.545	0.008204	0.0006	6.92567E-05	20.77700457	3.886130903
10	2264	L	M	4	0.7	0.009106	0.001034	0.00010753	32.25895014	4.697677318
11	2105	N	M	4	1.22	0.013112	0.0017129	0.000123708	37.11251664	3.100927177
12	2353	L	F	0	0.315	0.006264	0.0003946	5.96543E-05	17.89627598	5.791393938
13	2353	N	F	0	0.77	0.009782	0.0009809	9.49583E-05	28.48750488	3.771331252
14	1954	N	M	8	1.35	0.0124494	0.0018566	0.000141223	42.36695598	3.19907547
15	1954	L	M	8	0.705	0.008162	0.0008137	9.44069E-05	28.32207458	4.095122878
16	2264	N	F	8	1.2	0.016804	0.0018375	0.00010355	31.06504674	2.63889286
17	2264	L	F	8	0.65	0.019436	0.0011006	5.36239E-05	16.08718124	2.52288579
18	1819	L	M	8	1.1	0.020352	0.0016341	7.6034E-05	22.81018841	2.113815996

19	1819	N	M	8	1.648	0.018854	0.0025302	0.000127083	38.12489754	2.358209966
20	2321	L	F	4	0.5	0.00869	0.0006689	7.28916E-05	21.86748091	4.45820202
21	2321	N	F	4	1.38	0.018832	0.0020918	0.000105186	31.55593477	2.33094999
22	1870	L	F	4	0.975	0.017154	0.0010517	5.80581E-05	17.41741921	1.821000989
23	1870	N	F	4	1.65	0.021184	0.002689	0.000120204	36.0611997	2.227856529
24	1196	L	F	8	1	0.014958	0.0012842	8.13009E-05	24.39026243	2.486265284
25	1196	N	F	8	1.52	0.018002	0.0018223	9.58595E-05	28.75785266	1.928607534
26	2105	L	M	96	1.2	0.014758	0.0016274	0.000104425	31.32738484	2.661177781
27	1997	L	F	96	1.52	0.022518	0.0021488	9.03654E-05	27.10962543	1.818071344
28	1997	N	F	96	2.63	0.029892	0.0039573	0.000125366	37.6098272	1.45772829
29	2105	N	M	96	2.265	0.02643	0.0035553	0.000127384	38.21522547	1.719884223
30	2342	L	F	96	1.605	0.026844	0.0027033	9.53637E-05	28.60911021	1.817022506
31	2342	N	F	96	2.005	0.029216	0.0029106	9.43404E-05	28.30212897	1.438916926
32	2353	N	M	96	1.73	0.024948	0.0025446	9.65873E-05	28.97617954	1.707363581
