# Supplementary material for: Does intrauterine crowding affect the force generating capacity and muscle composition of the piglet front limb?
Source: PLoS One. 2019 Oct 10;14(10):e0223851. doi: 10.1371/journal.pone.0223851 (PMC6786600; doi:10.1371/journal.pone.0223851)
Supplement: S2 Table — (PDF) [file pone.0223851.s002.pdf]

GROUP MEANS (± SD) by CATEGORY

CATEGORY	BM (in kg)	SFLL (in m)	$F_{iso-max}$ (in N)	$F'_{iso-max}$
**L**	0.87 ± 0.37	0.17 ± 0.03	174.64 ± 52.70	21.99 ± 5.86
**N**	1.52 ± 0.46	0.21 ± 0.02	253.16 ± 44.50	17.68 ± 3.16

GROUP MEANS (± SD) by SEX

SEX	BM (in kg)	SFLL (in m)	$F_{iso-max}$ (in N)	$F'_{iso-max}$
**F**	1.25 ± 0.57	0.20 ± 0.03	217.16 ± 70.18	19.43 ± 5.71
**M**	1.18 ± 0.49	0.18 ± 0.03	215.31 ± 52.59	20.04 ± 4.23

GROUP MEANS (± SD) by AGE

AGE (in h)	BM (in kg)	SFLL (in m)	$F_{iso-max}$ (in N)	$F'_{iso-max}$
**0**	0.97 ± 0.44	0.19 ± 0.03	216.21 ± 80.38	23.79 ± 5.11
**4**	1.01 ± 0.41	0.18 ± 0.03	191.72 ± 61.39	20.28 ± 4.16
**8**	1.15 ± 0.36	0.19 ± 0.02	199.42 ± 38.84	18.61 ± 3.92
**96**	1.85 ± 0.48	0.21 ± 0.01	264.05 ± 36.53	15.01 ± 2.40

GROUP MEANS (± SD) by AGE, split by CATEGORY

	BM (in kg) CATEGORY		SFLL (in m) CATEGORY		$F_{iso-max}$ (in N) CATEGORY		$F'_{iso-max}$ CATEGORY	
AGE (in h)	L	N	L	N	L	N	L	N
**0**	0.64 ± 0.28	1.23 ± 0.36	0.16 ± 0.03	0.20 ± 0.02	174.81 ± 83.09	249.33 ± 68.25	27.56 ± 5.70	20.77 ± 1.51
**4**	0.68 ± 0.21	1.35 ± 0.23	0.15 ± 0.02	0.20 ± 0.02	140.91 ± 30.75	242.53 ± 31.03	22.09 ± 5.41	18.47 ± 1.54
**8**	0.86 ± 0.22	1.43 ± 0.20	0.18 ± 0.01	0.20 ± 0.01	167.11 ± 19.03	231.72 ± 19.36	20.43 ± 4.26	16.79 ± 2.96
**96**	1.44 ± 0.21	2.16 ± 0.38	0.20 ± 0.02	0.22 ± 0.01	229.42 ± 15.37	290.02 ± 20.32	16.48 ± 2.81	13.91 ± 1.57

GROUP MEANS (± SD) by AGE, split by SEX

	BM (in kg)		SFLL (in m)		$F_{iso\text{-}max}$ (in N)		$F'_{iso\text{-}max}$	
	SEX		SEX		SEX		SEX	
AGE (in h)	F	M	F	M	F	M	F	M
0	0.97 ± 0.47	0.97 ± 0.47	0.19 ± 0.04	0.18 ± 0.02	217.03 ± 91.47	214.57 ± 70.12	23.82 ± 5.93	23.74 ± 4.07
4	1.13 ± 0.50	0.90 ± 0.33	0.19 ± 0.03	0.17 ± 0.03	194.61 ± 80.16	188.84 ± 48.43	18.41 ± 4.09	22.16 ± 3.77
8	1.09 ± 0.36	1.20 ± 0.40	0.19 ± 0.01	0.19 ± 0.02	193.73 ± 35.49	205.10 ± 46.62	19.25 ± 5.40	17.97 ± 2.34
96	1.94 ± 0.51	1.73 ± 0.53	0.22 ± 0.01	0.21 ± 0.02	263.35 ± 44.94	264.97 ± 31.18	14.07 ± 1.32	16.26 ± 3.25

GROUP MEANS (± SD) by AGE, split by CATEGORY and SEX

	BM (in kg)				SFLL (in m)			
	CATEGORY				CATEGORY			
	L		N		L		N	
	SEX		SEX		SEX		SEX	
AGE (in h)	F	M	F	M	F	M	F	M
0	0.69 ± 0.33	0.53 (no SD)	1.25 ± 0.44	1.20 ± 0.37	0.17 ± 0.03	0.15 (no SD)	0.21 ± 0.03	0.19 ± 0.01
4	0.74 ± 0.34	0.63 ± 0.11	1.51 ± 0.19	1.18 ± 0.06	0.16 ± 0.02	0.14 ± 0.01	0.21 ± 0.01	0.19 ± 0.01
8	0.83 ± 0.25	0.90 ± 0.28	1.36 ± 0.27	1.50 ± 0.21	0.18 ± 0.01	0.18 ± 0.01	0.20 ± 0.003	0.21 ± 0.01
96	1.56 ± 0.06	1.2 (no SD)	2.32 ± 0.44	2.00 ± 0.38	0.21 ± 0.01	0.19 (no SD)	0.23 ± 0.01	0.22 ± 0.004

	$F_{iso\text{-}max}$ (in N)				$F'_{iso\text{-}max}$			
	CATEGORY				CATEGORY			
	L		N		L		N	
	SEX		SEX		SEX		SEX	
AGE (in h)	F	M	F	M	F	M	F	M
0	185.02 ± 98.64	144.17 (no SD)	249.03 ± 90.09	249.77 ± 49.00	27.42 ± 6.97	27.99 (no SD)	20.21 ± 0.64	21.61 ± 2.43
4	128.05 ± 13.78	153.77 ± 44.56	261.17 ± 36.98	223.90 ± 11.49	19.26 ± 6.87	24.92 ± 2.91	17.56 ± 0.28	19.40 ± 1.92
8	165.85 ± 1.96	168.38 ± 32.80	221.62 ± 25.8	241.82 ± 7.1	21.50 ± 6.69	19.37 ± 2.29	17.01 ± 4.76	16.57 ± 1.85
96	228.46 ± 21.60	231.34 (no SD)	298.25 ± 26.85	281.79 ± 15.73	14.89 ± 0.84	19.65 (no SD)	13.25 ± 1.35	14.57 ± 1.96
