# Supplementary material for: Does intrauterine crowding affect the force generating capacity and muscle composition of the piglet front limb?
Source: PLoS One. 2019 Oct 10;14(10):e0223851. doi: 10.1371/journal.pone.0223851 (PMC6786600; doi:10.1371/journal.pone.0223851)
Supplement: S3 Table — (PDF) [file pone.0223851.s003.pdf]

					TOTAL MASS		% TOTAL MASS		TOTAL MUSCLE MASS		% TOTAL MUSCLE MASS	
PIG	SEX	AGE (in h)	BW/V	BM (in g)	FRONT LIMB	HIND LIMB	FRONT LIMB	HIND LIMB	FRONT LIMB	HIND LIMB	FRONT LIMB	HIND LIMB
1	M	0	L	525	26.29	25.98	5.01	4.95	14.76	15.86	2.81	3.02
2	M	0	N	940	52.72	60.55	5.61	6.44	29.93	39.12	3.18	4.16
3	F	0	N	1370	68.57	81.92	5.01	5.98	37.29	54.01	2.72	3.94
4	F	0	N	1620	93.38	106.26	5.76	6.56	51.66	68.21	3.19	4.21
5	F	0	L	795	38.57	40.36	4.85	5.08	21.93	24.83	2.76	3.12
6	M	0	N	1458	76.70	84.78	5.26	5.81	43.20	52.62	2.96	3.61
7	M	4	N	1140	58.86	67.27	5.16	5.90	32.92	42.45	2.89	3.72
8	F	0	L	955	49.61	54.50	5.20	5.71	26.47	32.71	2.77	3.43
9	M	4	L	545	23.16	23.87	4.25	4.38	12.57	13.94	2.31	2.56
10	M	4	L	700	34.79	35.91	4.97	5.13	20.44	23.43	2.92	3.35
11	M	4	N	1220	60.74	70.55	4.98	5.78	33.53	45.38	2.75	3.72
12	F	0	L	315	14.54	14.36	4.62	4.56	7.19	8.49	2.28	2.69
13	F	0	N	770	38.10	40.14	4.95	5.21	19.35	24.38	2.51	3.17
14	M	8	N	1350	63.36	67.08	4.69	4.97	34.75	43.60	2.57	3.23
15	M	8	L	705	30.85	31.73	4.38	4.50	16.36	19.33	2.32	2.74
16	F	8	N	1200	69.70	86.21	5.81	7.18	38.44	58.10	3.20	4.84
17	F	8	L	650	42.69	60.68	6.57	9.34	22.63	32.53	3.48	5.00
18	M	8	L	1100	58.95	68.10	5.36	6.19	33.49	44.96	3.04	4.09
19	M	8	N	1648	86.43	105.49	5.24	6.40	47.88	67.45	2.91	4.09
20	F	4	L	500	24.07	23.31	4.81	4.66	12.71	13.41	2.54	2.68
21	F	4	N	1380	74.50	83.76	5.40	6.07	41.01	52.66	2.97	3.82
22	F	4	L	975	44.71	49.77	4.59	5.10	22.18	29.16	2.28	2.99
23	F	4	N	1650	99.90	114.15	6.05	6.92	52.99	70.18	3.21	4.25
24	F	8	L	1000	46.00	50.33	4.60	5.03	24.86	31.09	2.49	3.11
25	F	8	N	1520	66.06	84.79	4.35	5.58	35.04	55.83	2.31	3.67
26	M	96	L	1200	55.25	61.08	4.60	5.09	32.18	39.07	2.68	3.26
27	F	96	L	1520	68.97	91.42	4.54	6.01	38.37	60.02	2.52	3.95
28	F	96	N	2630	136.11	166.27	5.18	6.32	79.70	112.67	3.03	4.28
29	M	96	N	2265	115.06	136.34	5.08	6.02	69.20	90.65	3.06	4.00
30	F	96	L	1605	87.96	103.93	5.48	6.48	48.01	65.92	2.99	4.11
31	F	96	N	2005	100.52	131.32	5.01	6.55	55.88	83.75	2.79	4.18

32	M	96	N	1730	86.71	89.72	5.01	5.19	50.24	54.06	2.90	3.12

PIG	SEX	AGE (in h	BW/V	BM (in g)	SELECTED MUSCLE MASS		% SELECTED MUSCLE MASS	
1	M	0	L	525	FRONT LIMB	HIND LIMB	FRONT LIMB	HIND LIMB
2	M	0	N	940	7.82	11.44	1.49	2.18
3	F	0	N	1370	15.58	29.92	1.66	3.18
4	F	0	N	1620	20.13	39.62	1.47	2.89
5	F	0	L	795	27.46	51.38	1.70	3.17
6	M	0	N	1458	11.15	18.40	1.40	2.32
7	M	4	N	1140	21.00	40.38	1.44	2.77
8	F	0	L	955	16.52	31.77	1.45	2.79
9	M	4	L	545	13.47	24.98	1.41	2.62
10	M	4	L	700	6.33	10.24	1.16	1.88
11	M	4	N	1220	10.59	17.43	1.51	2.49
12	F	0	L	315	16.81	34.31	1.38	2.81
13	F	0	N	770	3.58	6.26	1.14	1.99
14	M	8	N	1350	9.94	18.35	1.29	2.38
15	M	8	L	705	17.84	32.64	1.32	2.42
16	F	8	N	1200	8.31	13.52	1.18	1.92
17	F	8	L	650	19.93	43.33	1.66	3.61
18	M	8	L	1100	11.61	24.18	1.79	3.72
19	M	8	N	1648	16.26	33.32	1.48	3.03
20	F	4	L	500	26.01	51.56	1.58	3.13
21	F	4	N	1380	6.12	10.24	1.22	2.05
22	F	4	L	975	21.18	39.69	1.54	2.88
23	F	4	N	1650	11.37	21.90	1.17	2.25
24	F	8	L	1000	26.79	53.19	1.62	3.22
25	F	8	N	1520	12.69	23.66	1.27	2.37
26	M	96	L	1200	17.83	41.56	1.17	2.73
27	F	96	L	1520	16.28	29.07	1.36	2.42
28	F	96	N	2630	18.98	44.55	1.25	2.93

29	M	96	N	2265	40.20	84.16	1.53	3.20
30	F	96	L	1605	36.93	69.24	1.63	3.06
31	F	96	N	2005	24.80	49.14	1.55	3.06
32	M	96	N	1730	28.07	63.27	1.40	3.16
					25.96	40.74	1.50	2.35
